# Advanced Glycation End Products: Link between Diet and Ovulatory Dysfunction in PCOS?

**DOI:** 10.3390/nu7125524

**Published:** 2015-12-04

**Authors:** Deepika Garg, Zaher Merhi

**Affiliations:** 1 Department of Obstetrics and Gynecology, Maimonides Medical Center, Brooklyn, NY 11219, USA; drgargdeepika@gmail.com; 2 Department of Obstetrics and Gynecology, Division of Reproductive Biology, School of Medicine, New York University, 180 Varick St., Sixth Floor, New York, NY 11014, USA

**Keywords:** advanced glycation end products, receptor for advanced glycation endproducts (RAGE), soluble receptor for advanced glycation end products (sRAGE), polycystic ovary syndrome (PCOS), anovulation

## Abstract

PCOS is the most common cause of anovulation in reproductive-aged women with 70% experiencing ovulatory problems. Advanced glycation end products are highly reactive molecules that are formed by non-enzymatic reactions of sugars with proteins, nucleic acids and lipids. AGEs are also present in a variety of diet where substantial increase in AGEs can result due to thermal processing and modifications of food. Elevation in bodily AGEs, produced endogenously or absorbed exogenously from high-AGE diets, is further exaggerated in women with PCOS and is associated with ovulatory dysfunction. Additionally, increased expression of AGEs as pro-inflammatory receptors in the ovarian tissue has been observed in women with PCOS. In this review, we summarize the role of dietary AGEs as mediators of metabolic and reproductive alterations in PCOS. Once a mechanistic understanding of the relationship between AGEs and anovulation is established, there is a promise that such knowledge will contribute to the subsequent development of targeted pharmacological therapies that will treat anovulation and improve ovarian health in women with PCOS.

## 1. Introduction

PCOS is an endocrine disorder that is characterized by hyperandrogenism, oligo/anovulation and polycystic ovaries and it affects up to 25% of reproductive-aged women [1]. Although the exact pathophysiology of PCOS is still unknown, environmental and genetic factors play an important role in the clinical phenotypes, including ovulatory dysfunction, of PCOS. Diet with high advanced glycation end products (AGEs) content represents one of the environmental factors that are related to both the metabolic and reproductive alterations observed in PCOS [2].

AGEs are a diverse group of reactive molecules that are formed endogenously by non-enzymatic reactions of carbonyl group of carbohydrates with free amino groups of proteins, nucleic acids or lipids [3]. This glycoxidation reaction is called browning or Maillard reaction [4]. In addition to their endogenous formation, AGEs exist in high amounts in cooked fast-food diet [5]. Elevated serum AGEs levels have been observed in patients with hyperglycemia, insulin resistance, diabetes, renal insufficiency, atherosclerosis, aging, rheumatoid arthritis and recently PCOS [6,7,8]. These high circulating levels of AGEs can cause cellular damage after deposition in different tissues [9].

**Figure 1:**
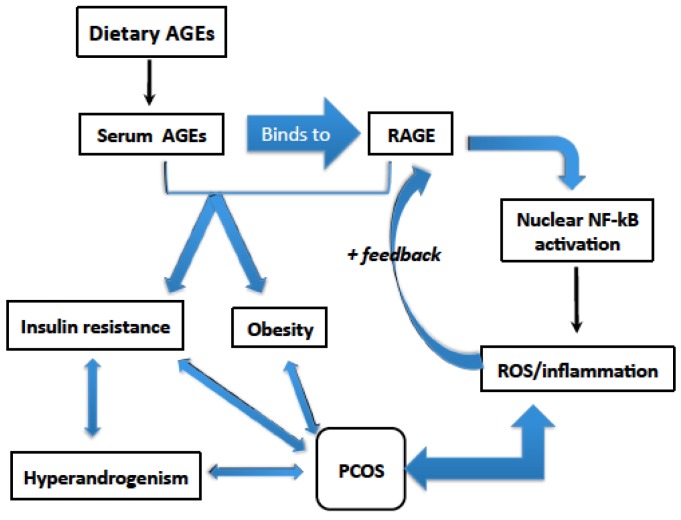
Possible Roles of dietary AGEs in PCOS.

Recent data have shown that AGEs’ circulating levels and expression of their pro-inflammatory receptors in the ovarian tissue called as receptor for advanced glycation end products (RAGE) are elevated in women with PCOS [10]. On the other hand, high levels of the protective anti-inflammatory receptors called soluble receptor for advanced glycation end products (sRAGE) are associated with protection against AGEs [11]. Interestingly, studies have demonstrated that the intake of low-AGE containing diet is associated with favorable metabolic and hormonal profile as well as less oxidative stress biomarkers in patients with PCOS [12]. In this review, we will report the relationship between dietary AGEs and PCOS phenotypes, with a focus on the role of dietary AGEs in ovulatory dysfunction, obesity, insulin resistance, and hyperandrogenism (Figure 1, Table 1).

**Table 1:** Role of dietary AGEs in metabolic and hormonal dysfunction.

Study	Subjects, Animals, or Cell Lines	Intervention	Outcome
Leuner B. *et al.*, 2012 [13]	C57BL/6 mice	RAGE+ or RAGE− mice given high-fat diet to induce obesity	RAGE- mice had high insulin levels and accelerated weight gain
Cai W. *et al.*, 2012 [14]	C57BL6 mice	Isocaloric diet with or without synthetic MG	Mice given MG developed premature insulin resistance, adiposity and the inflammatory changes
Hofmann S.M. *et al.*, 2002 [15]	C57/BL/KsJ db/db female mice	High-AGE *vs.* low-AGE diet	Low-AGE diet: lower fasting insulin levels, reduction in body weight, improved glucose and insulin tolerance tests, increased plasma HDL and lower serum CML and MG levels
Sandu O. *et al.*, 2005 [16]	C57/BL6 and db/db (++) female mice	High-AGE *vs.* low-AGE diet	High-AGE diet: elevated insulin levels, change in pancreatic islet structure
Chatzigeorgiou A. *et al.*, 2013 [17]	female wistar rats	High-AGE *vs.* low-AGE diet	High-AGE diet: increased glucose, insulin, and testosterone levels
Cassese A. *et al.*, 2008 [18]	C57/BL6 female mice	High-AGE *vs.* low-AGE diet	High-AGE diet: increase in insulin resistance and impairment in insulin sensitivity
Kandaraki E. *et al.*, 2012 [19]	female Wistar rats	High-AGE *vs.* low-AGE diet	High-AGE diet: reduced ovarian GLO-I activity and high AGE expression in the granulosa cells
Diamanti-Kandarakis *et al.*, 2007 [20]	Female rats	High-AGE *vs.* low-AGE diet	High-AGE diet: high fasting glucose, insulin, testosterone, and serum AGEs; higher AGE localization in the theca interna cells; elevated RAGE expression in granulosa cells
Gaens K.H. *et al.*, 2014 [21]	Obese RAGE- *vs.* RAGE + mice	Measured CML in plasma and adipose tissue	RAGE+: reduced plasma CML level with entrapment in the adipose tissue, altered inflammatory profile and glucose homeostasis
Diamanti-Kandarakis E. *et al*., 2015 [22]	KGN: human granulosa cell line	Culture was done with HGA or insulin or both HGA + insulin	Altered insulin signaling and Glut-4 translocation after HGA exposure
Diamanti-Kandarakis E. *et al.*, 2008 [23]	Young lean non-insulin resistant women with PCOS *vs.* healthy women and *vs.* women with isolated features of PCOS	Measurement of serum AGEs	Elevated levels of AGEs in young lean non-insulin resistant women with PCOS
Mark A.B. *et al.*, 2014 [24]	Overweight women	High-AGE *vs.* low-AGE diet	Low-AGE diet: lower fasting insulin levels, urinary AGEs, and insulin resistance
Tantalaki E. *et al.*, 2014 [12]	Women with PCOS	High-AGE *vs.* low-AGE diet	Low-AGE diet: reduction in insulin level and HOMA
Diamanti-Kandarakis *et al.*, 2009 [25]	Women with or without PCOS	Measured serum AMH and AGEs	Higher AMH and AGEs in PCOS women with ovulatory dysfunction. Positive correlation with AMH/AGEs ratio to number of follicles
Diamanti-Kandarakis E. *et al*., 2007 [10]	Women with or without PCOS	AGE and RAGE immunoreactivity	PCOS: higher AGE and RAGE immunoexpression in granulosa cells
Diamanti-Kandarakis *et al.*, 2005 [26]	Women with or without PCOS	Measured serum AGE levels and RAGE expression in circulating monocytes	PCOS: higher AGEs’ levels with increased RAGE expression, higher testosterone and free androgen index (FAI), waist-to-hip ratio and HOMA
Gaens K.H. *et al.*, 2014 [21]	Human preadipocytes	Measured CML levels and RAGE expression	The activation of AGE-RAGE axis is involved in the dysregulation of adipokines in obesity, thereby contributing to the development of obesity-associated insulin resistance

RAGE = receptors of advanced glycation end products; MG = methylglyoxal; AGE = Advanced Glycation End Products; HDL = high density lipoprotein; CML = *N*-carboxymethyl-lysine; PCOS = polycystic ovary syndrome; HOMA = Homeostasis Model Assessment index; GLO-I = Glyoxalase 1; d-gal = d-galactose; AMH = anti-mullerian hormone; HGA = human glycated albumin.

## 2. Dietary AGEs and Their Receptors

Endogenously, AGEs are the results of post-translational modifications of non-enzymatic advanced glycation and oxidation (glycoxidation) reactions of amino peptides, nucleic acids and lipids by glucose [27,28]. AGEs constitute a family of more than 20 members of heterogeneous group of compounds such as *N*-carboxymethyl-lysine (CML), pentosidine and reactive intermediates like methylglyoxal (MG). Pentosidine and CML are some of the most commonly studied AGEs [20]. In addition to the endogenous AGEs, exogenously derived sources of AGEs include tobacco and certain types of food in modern diet [29,30]. Uncooked animal-derived foods are the major source of AGEs and thermal processing accelerate AGEs’ formation [4]. The ultimate consumption of AGE-rich diet leads to their absorption through the gastrointestinal system [31]. Diet containing high protein and fat has higher amount of the AGEs, such as CML and MG, in comparison to carbohydrate-rich diet [32]. Cooking methods also affect dietary AGEs content in a diet such as food prepared at low temperature with high moisture with brief heating time have less AGEs and also use of acidic marinades such as lemon juice and vinegar during cooking significantly reduces the AGE’s content in the diet [32].

The highest level of AGEs per gram of food is present in dry-heat processed foods such as chips, crackers, and cookies, which is due to the presence of oil, butter, cheese, nuts and eggs as ingredients in these foods [33]. Dry-heat processing also accelerates dietary AGEs’ formation in lean red meats and poultry due to presence of reactive amino-lipids and reducing sugars (fructose as well as glucose-6- phosphate) [34]. Full-fat American and Parmesan cheeses contain more dietary AGEs in comparison to mozzarella, cottage and 2% milk cheddar cheeses; this is most likely due to pasteurization and long holding times at room temperatures and continued slow glycoxidation reactions [35]. Fruits, vegetables, low fat milk, grains and legumes have the lowest dietary AGEs’ content [5]. Milk products such as yogurt, pudding, and ice cream contain low levels of AGEs due to high moisture index [32].

### 2.1. Dietary AGEs Activate the Pro-Inflammatory Receptor RAGE

AGEs can act by direct cross linkage with extracellular membrane (receptor independent) or by interacting with their receptors called RAGE that are present on the cell surfaces of many tissues. RAGE is a transmembrane receptor that belongs to the immunoglobulin superfamily [36]. The interaction of AGE-RAGE leads to the activation of pro-inflammatory and oxidative stress cascades such as Nuclear Factor Kappa B (NF-κB) by inducing the expression of several pro-inflammatory genes, thus leading to tissue damage [3,37,38]. RAGE is expressed in various tissues throughout the body including lung, heart, blood vessel wall, skeletal muscles, ovaries, and inflammatory cells such as monocytes, macrophages and lymphocytes [11]. AGE-RAGE binding induces RAGE expression thus forming a positive feedback loop between the ligand and its receptor [23,37].

### 2.2. Soluble Receptor for AGEs (sRAGE) Protects against Dietary AGEs

A soluble form of RAGE described as soluble C-truncated RAGE (sRAGE) lacks both transmembrane and cytosolic domains of RAGE and formed after alternative splicing of *RAGE* gene [39,40]. The sRAGE receptor circulates throughout the body and forms a decoy receptor that binds circulating AGEs, thus preventing them from interacting with their pro-inflammatory RAGE receptor ultimately preventing tissue damage [41]. Although it remains contentious, a reduced sRAGE level indicates a heightened RAGE signaling and more pathologies. A protective role for sRAGE has been identified in animal models in various RAGE-mediated disorders such as type II diabetes and wound healing [42,43]. Additionally, sRAGE has been shown to neutralize the action of AGEs and was found to exert a protective role against cardiovascular disease [44]. In a recent study, sRAGE concentration in ovarian follicular fluid was positively correlated with embryo quality and In Vitro Fertilization (IVF) outcome [45].

## 3. Dietary AGEs Induce Inflammation in PCOS

**Figure 2:**
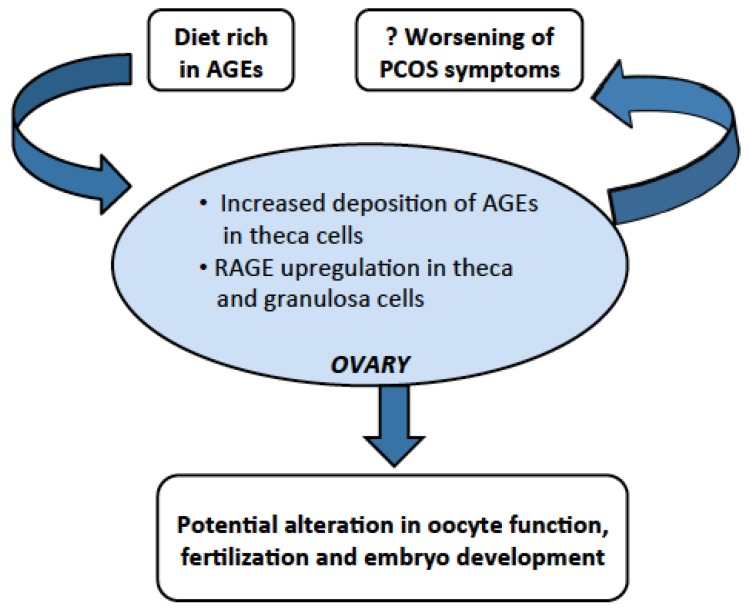
Possible involvement of AGEs in ovulatory dysfunction in PCOS.

The dietary intake of AGEs lead to elevated AGEs’ serum levels which has been shown to correlate with inflammatory markers such as high sensitivity C-reactive protein (CRP), fibrinogen, 8-isoprostanes (a marker of lipid peroxidation), tumor necrosis factor-alpha (TNF-α), vascular adhesion molecule-1 (VAM-1) and Homeostasis Model Assessment index (HOMA) an indicator of insulin resistance [46,47] (Figure 2). Dietary AGEs also have similar cell activating and cell oxidative capabilities as endogenous AGEs thus inducing inflammatory signals and promoting oxidative stress [46,47]. In addition, accumulation of dietary AGEs in the tissues further enhances cellular damage via production of reactive oxygen species [48]. One of the hallmarks of PCOS is chronic low-grade inflammation, which has been reported as partly responsible for the metabolic and reproductive dysfunction associated with PCOS [49]. Whether dietary AGEs directly contribute and/or exaggerate this inflammatory state observed in PCOS needs to be determined in future studies since studies to date showed only a correlation.

After consumption of high-AGE diets, elevation in serum AGEs level occurs in direct proportion of the ingested AGEs [31]. Approximately, 10% of orally absorbed AGEs remain in the bioactive form [31]. Vlassara H *et al.* [46] compared the effect of high-AGE diet *vs.* 5-fold lower AGE content diet on serum AGEs level and inflammatory mediators in non-smoking diabetic patients of age group of 52 to 62 years. They reported a statistically significant 64.5% increase in serum AGEs in patients on high-AGE diet and a statistically significant 30% decrease in serum AGEs in patients on low-AGE diet (*p* = 0.02). High-AGE diet was also associated with a statistically significant 86.3% elevation in TNF-α (*p* = 0.006), a statistically significant 35% elevation in CRP (*p* = 0.014), and a 4% rise in VAM-1 (*p* = 0.01) when compared with low-AGE diet [46]. Uribarri *et al.* [47] investigated the serum AGE levels and the inflammatory and oxidative response after high intake of dietary AGEs in 172 (102 women and 70 men) healthy volunteers divided in two age groups (116 aged between 18 to 45 years, 56 aged between 60 to 80 years) [47]. Dietary intake of AGEs was significantly correlated with the level of circulating AGEs (CML and MG; *p* < 0.05 for both) and with serum CRP levels (*p* = 0.04) [47].

Glyoxalase 1 (GLO-1), a physiologic defense enzyme against glycation, is found in processed fatty food [50]. GLO-1 metabolizes MG thus providing protection against AGEs [50]. Higher levels of GLO-1 are associated with low AGE levels and lower oxidative stress in diabetic GLO-I transgenic rats animal model in comparison to diabetic wild-type rats and GLO-1-overexpressing mesangial cells which were cultured in high glucose to mimic diabetic conditions [51,52]. In PCOS, elevated levels of AGEs can occur due to reduced bodily activity of GLO-1. Kandaraki E *et al.* [19] showed that dietary AGEs and excessive androgens in ovaries reduce the activity of GLO-1 leading to the accumulation of AGEs in ovarian tissues in female Wistar rats and causing the formation of reactive oxygen species (ROS); all this leading to ovarian dysfunction. They proposed the mechanism for worsening ovulatory dysfunction in PCOS, which is a hyperandrogenic state, is due to reduced GLO-1 activity by dietary AGEs leading to increase in AGEs deposition and elevated inflammatory markers in ovaries [19]. Taken together, it is highly plausible that consumption of high-AGE diet contributes to a sustained low-grade inflammatory state associated with PCOS.

## 4. Dietary AGEs Cause Ovarian Dysfunction in PCOS

It has been documented that the deposition of AGEs in the ovaries interfere with the physiological remodeling during folliculogenesis and negatively impact oocyte maturation, development and chromosomal composition in the ovaries [53]. In PCOS, there is elevation in intraovarian AGEs’ deposition and there is RAGE upregulation in granulosa cells [10] (Figure 2). Similar findings were reported by Diamanti-Kandarakis *et al.* [20] who investigated the presence of dietary AGEs in ovarian tissue and their role in metabolic and hormonal dysfunctions in rats. In that study, they assigned rats to high-AGE or low-AGE diets for 6 months [20]. Animals on high-AGE diet had increased deposition of AGEs in their theca cells, increase expression of RAGE in theca interna and granulosa cells, elevated fasting glucose, insulin, serum AGEs, and plasma testosterone levels compared to animals on low-AGE diet [20]. In a study, inhibition of AGE-RAGE system in d-galactose (d-gal)-induced animal model showed correction of elevated levels of AMH, total testosterone and abnormal estrous cycles thus improving the ovulatory dysfunction in this animal model in PCOS [54]. Recently, Tantalaki *et al.* [12] studied the role of diet containing AGEs and its effect on metabolic and hormonal derangements in PCOS. An isocaloric diet containing high-AGE content *vs.* low-AGE content was given to 23 women with PCOS and changes in metabolic, hormonal and oxidative stresses were assessed. They concluded that the diet containing low AGEs along with the life style modifications was beneficial to patients with PCOS [12].

The significance of dicarbonyl stress by deposition of AGEs in the ovarian tissue has been established in recent data [10]. The role of AGEs has been defined as key agents in affecting the ovarian function by altering oocyte growth and development [53]. It is reasonable to speculate that dietary AGEs are responsible for high levels of serum and intraovarian AGEs concentration in PCOS, which may be responsible for the ovulatory dysfunction by elevation of AGEs’ metabolite, in particular MG. The passage of MG to oocyte through cumulus cells, may pose a significant risk during oocyte development by affecting the genome integrity. MG also alters mitochondrial function, and triggers apoptosis and DNA damage within the oocyte [55]. Additionally, the role of elevated MG levels in altering oocyte development, fertilization, and embryonal growth has been evaluated in mouse model [56]. During the oocyte development, molecular and energetic supply largely depends on the glycolysis in cumulus cells of the cumulus-oocyte complex and follicular fluid as the oocyte is not able to metabolize glucose itself [57]. Pyruvate, along with other byproduct of glycolysis such as MG is transferred to oocyte for oxidative phosphorylation from cumulus cells. Based on these findings, it can be postulated that the intermediate byproducts of dietary AGEs such as MG might affect oocyte formation and development by similar mechanism; however this is an area, which needs further exploration.

In addition to elevated AGEs and RAGE expression in granulosa cells of the polycystic ovaries, another mechanism of AGEs induced ovulatory dysfunction in PCOS is proposed via interference in sustained LH action via AGE-RAGE signaling which leads to ERK1/2 MAPKs activation (mitogen-activated protein kinase) during oocyte maturation [58]. Luteinizing hormone (LH) induces oocyte maturation in preovulatory follicles by regulation of MAPK in granulosa cells [59]. Diamanti-Kandarakis E *et al.* [58] investigated the activation of ERK1/ERK2 MAPKs in human ovarian granulosa cell line model (KGN) after culture with human glycated albumin (HGA, as a source of AGEs) and reported 2.5 times elevation in p-ERK1/2 levels which was suppressed within 2 h interfering with sustained activation ERK1/2 in KGN cells. Their results suggested that HGA affect normal follicle development and ovulation process in a pattern similar to that observed in PCOS.

The main pathophysiological mechanisms associated with ovulatory dysfunction in PCOS is due to increased number of small antral follicles due to defective selection of the dominant follicle which leads to increased level of AMH [60]. AMH is produced by granulosa cells of primary follicles, expressed in small antral follicles and plays an important role in dominant follicle selection [61]. In PCOS, AMH levels are abnormally elevated [62]. Diamanti-Kandarakis *et al.* [25] investigated the association between serum levels of AMH and AGEs in women with and without PCOS. The results showed statistically significantly higher AMH and AGEs levels in anovulatory women with PCOS and there was a significant positive correlation between AMH and AGEs (*p* < 0.01) [25]. These findings suggest that the elevated serum AGEs are associated with elevated AMH and with anovulation observed in PCOS.

## 5. Dietary AGEs Are Associated with Insulin Resistance in PCOS

Insulin resistance is a well-established principle contributor to the underlying pathophysiology of PCOS [63,64]. Insulin resistance is present in around 50%–70% women with PCOS [65]. Consumption of diet containing low AGEs has been shown to improve insulin sensitivity and wound healing in mice [15,66]. Higher insulin levels were noted in C57BL/6 RAGE −/− mice which were fed a diet containing high fat to induce obesity when compared with RAGE +/+ animals [13]. Recently, the role of dietary AGEs in the development of insulin resistance and type II diabetes has been proposed in a study by Cai *et al.* [14] where isocaloric diet with or without synthetic MG (+) was fed to the C57BL6 mice. MG (+) mice developed premature insulin resistance, adiposity and the inflammatory phenotypic shift to the homeostatic environment then MG (−) mice [14]. Hofmann *et al.* [15] studied the role of dietary AGEs in insulin resistance in 4 weeks old C57/BL/KsJ db/db mice that were divided in two groups and were placed on either low- or high-AGE diet for 20 weeks. Animal on low-AGE diet had statistically significant lower fasting insulin levels, 13% reduction in body weight, improved glucose and insulin tolerance tests, increased plasma HDL levels and lower serum CML and MG levels compared to animals on high-AGE diet [15].

In another study, consumption of diet containing high-AGE content was linked to the development of insulin resistance in C57/BL6 mice [16]. Sandu O *et al.* [16] studied the role of AGEs in insulin resistance in C57/BL6 and db/db mice that were fed low- or high-AGE diet for 6 months and results showed elevated insulin levels (*p* < 0.001) with change in pancreatic islet structure in high-AGE fed animals as compared to low-AGE fed animals [16]. In a recent experiment, increased glucose, insulin and testosterone levels were found in high-AGE fed female Wistar rats along with significant down- regulation of RAGE expression (*p* = 0.041) which was negatively correlated with serum insulin and testosterone levels signifying the role of diet rich is AGEs in the development of insulin resistance [17].

Diamanti-Kandarakis *et al.* [22] studied the effects of AGEs on intracellular insulin signaling pathways and transport of glucose in human granulosa KGN cell line. They found that AGEs interact with insulin signaling pathways and interfere with glucose transport in KGN cells which can lead to development of insulin resistance and ovulatory dysfunction in conditions like PCOS [22]. In another study, Diamanti-Kandarakis *et al.* [67] investigated serum levels of AGEs, sex hormones, fasting glucose and insulin in postmenopausal women and found statistically significant higher serum AGEs levels (*p* < 0.0005) in women with high testosterone levels and higher free androgen index after adjusting for age, BMI, fasting glucose and fasting insulin levels.

Insulin resistance is associated with oxidative stress and inflammation. The role of AGE-RAGE system and its downstream signaling has been identified in the pathogenesis of insulin resistance via inflammatory response [68,69]. Recently, increased expression of RAGE and AGE-modified proteins has been found in granulosa cells of ovarian tissue in women with PCOS [10]. It is plausible that increased AGE-RAGE expression in women with PCOS could play a role in the pathogenesis of insulin resistance associated with PCOS. Direct association of the AGE-RAGE system has been found with insulin resistance independent to body weight and serum glucose levels [14]. In contrast, significantly higher levels of AGEs were found in young lean non-insulin resistant women who were diagnosed with PCOS (*p* < 0.001) in comparison to healthy women and women with isolated features of PCOS signifying elevated AGEs levels in PCOS without the presence of insulin resistance [23].

Mark *et al.* [24] investigated the effect of dietary AGEs on insulin sensitivity in overweight women. They fed 74 overweight women either low- or high-AGE diet and demonstrated a reduction in fasting insulin levels, urinary AGEs, and insulin resistance in women given low-AGE diet [24]. Tantalaki E *et al.* [12] investigated the role of dietary AGEs on serum levels of insulin and HOMA-IR index in women with PCOS who were fed high-AGE and low-AGE diet for 2 months and results showed a marked reduction in insulin and HOMA with low-AGE diet (*p* = 0.02 and *p* = 0.03 respectively) and strong positive correlation of dietary AGEs with insulin levels (*p* = 0.04). Emphasis has been given to modify the diet to avoid intake of AGEs and thus prevention of oxidative stress, which significantly improves insulin resistance in women with PCOS [70].

Insulin resistance also contributes to hyperandrogenism linked with PCOS and fasting insulin levels are correlated with androgen concentration as insulin is a strong stimulator of ovarian androgen production via the insulin receptors [71]. Cassese A *et al.* [18] found in their experiments that AGEs were able to induce insulin resistance and impairment in insulin sensitivity in C57/BL6 mice that were provided high-AGE diet. In patients with PCOS, RAGE levels are elevated (*p* = 0.02) and correlate with androgen levels (*p* = 0.007) further suggesting a role of the AGE/RAGE system in PCOS [72].

## 6. Dietary AGEs are Associated with Obesity in PCOS

Obesity plays a key role in the pathogenesis of PCOS with a prevalence of 30%–75% in women with PCOS [73]. Hofmann *et al.* [15] demonstrated that low-AGE fed C57/BL/KsJ db/db mice considerably lost weight by week 14 in comparison to high-AGE fed mice (*p* = 0.001), indicating a role for high-AGE diet in adiposity. Despite the association of obesity with PCOS, the knowledge about the diet content and meal patterns in women with PCOS remains sparse. Different studies showed conflicting data pertaining to normal or altered eating habits in women with PCOS [74,75]. In a recent study, the authors described the dietary intake patterns and attitudes in women with PCOS and showed higher intake of carbohydrates [75]. Leuner B *et al.* [13] analyzed insulin levels along with the change in body weight in RAGE +/+ or RAGE −/− C57BL/6 mice. They found that RAGE −/− mice had lower levels of AGEs in comparison to RAGE +/+ animals and they demonstrated that the absence of RAGE (RAGE −/−) was associated with weight gain and elevated insulin levels [13]. This could be explained by the role of insulin resistance and elevated insulin level in RAGE −/− mice leading to change in energy expenditure which caused the weight gain in these animals [74]. Intake of higher energy diet in women with PCOS along with lower resting energy expenditure in these women may contribute to weight gain independent of other factors, such as insulin resistance [76].

Involvement of AGE-RAGE system in the development of obesity has been suggested by current studies [16]. When C57/BL6 and db/db mice were fed low- or high-AGE diet, a significant increase in body weight (*p* < 0.001) was noted after 6 months in the high-AGE fed group compared to the low-AGE fed group [16]. In another study, C57BL6 mice were fed isocaloric diet with or without the AGE (MG). Animals who received high-MG diet showed increased adiposity along with pro-inflammatory changes in the adipocytes suggesting a role for AGEs in the development of obesity [14]. Another mechanism by which AGE-induced obesity was proposed was by suppression of survival factor called sirtuin 1 (SIRT1), this factor helps in mobilization of fatty acids and it was lower in mice on high-MG diet [14,77]. Additionally, Jia X *et al.* [78] investigated the association of MG, an intermediate in the formation of AGEs, with obesity in adipose tissue derived cell line 3T3-L1. They proposed that the possible mechanism for MG-induced obesity was by phosphorylation of PI3K/Akt signaling pathway and its target genes that caused proliferation of of 3T3-L1 cells [78].

Glyoxalase (GLO-I) is a ubiquitous enzyme and protects against AGEs induced cellular damage [50]. Recently, the effect of dietary AGEs was studied on the activity of this enzyme and weight gain in normal non-androgenized and androgenized prepubertal rats after feeding them high- or low-AGE diet for 3 months. The results of that study showed that markedly reduced GLO-1 activity in androgenized prepubertal rats on high-AGE diet in comparison to normal non-androgenized rats on high-AGE diet with higher weight gain (*p* < 0.001) in non-androgenized rats on low-AGE diet in comparison to androgenized rats on the same diet [19].

Studies in diabetic and non-diabetic subjects showed that among all the components of metabolic syndrome, BMI was a major risk factor inversely regulating serum sRAGE levels, the protective soluble receptor against AGEs, suggesting a relationship between adiposity and AGE-RAGE system [40]. Diamanti-Kandarakis *et al.* [26] demonstrated a significant positive correlation between serum AGEs level and waist-to-hip ratio (*p* < 0.02) in women with PCOS. Additionally, Gaen *et al.*, showed that RAGE-mediated CML (one of the AGEs) accumulation in human adipose tissue and the activation of the CML-RAGE axis caused dysregulation of adipokines in obesity [21]. Further, RAGE deficiency was associated with decreased fat mass and smaller adipocyte size, less body weight, and less epidermal fat weight [79,80]. Several studies demonstrated that plasma sRAGE level is inversely correlated with obesity [81,82]. Interestingly, sRAGE levels rise significantly after surgical weight loss, [83] further suggesting an association between AGEs and adiposity.

## 7. Reduced Intake of Dietary AGEs and Inhibition of the AGE-RAGE System

Dietary AGEs may modify both metabolic and hormonal parameters of affected women, without changes in BMI [12]. Diet modification has been shown to alter PCOS phenotypes; for instance, polyunsaturated fatty acids (PUFA) modulated hormonal and lipid profiles and supplementation with long-chain (LC) *n*-3 (omega-3) PUFA improved androgenic profiles in women with PCOS where LC *n*-3 PUFA supplementation reduced plasma bioavailable testosterone concentrations [84]. Additionally, Omega-3 fatty acids have been shown to improve insulin sensitivity in PCOS patients [85]. Reduced intake of diet containing AGEs showed less oxidative stress, improved insulin sensitivity and longer life span [86]. Moreover, diet-induced elevation of serum AGEs and the increased oxidative stress may directly induce insulin signaling defects [18]. Short and long-term food restrictions have shown to reduce the accumulation of AGEs [87,88].

As mentioned earlier, sRAGE provides protection against the deleterious effects of AGEs via binding them in the circulation and preventing their deposition in different tissues [89]. Therefore, one way to prevent the adverse impact of AGEs is by inducing the production of sRAGE. Interestingly, dietary vitamin D has shown to upregulate sRAGE levels and to reduce the deposition of AGEs and RAGE in various tissues [90,91,92]. In vitamin d-deficient women with PCOS, we [89] studied the role of vitamin D supplementation on serum sRAGE levels and we found a statistically significant increase in serum sRAGE (*p* = 0.03). One limitation of our study is that we did not evaluate the relationship between the changes in serum sRAGE and ovulatory function in women with PCOS.

AGE-RAGE inhibitors can also represent a potential therapeutic option for ovulatory dysfunction in women with PCOS. These inhibitors can be either natural or synthetic compounds and inhibit AGE-induced tissue damage via different mechanisms [93,94,95,96] (Table 2). Some of these compounds slow down the absorption of dietary AGEs [97]. Aminoguanidine, one of the AGE inhibitors, has been evaluated in d-galactose induced PCO-animal model where PCO-like phenotype was achieved by daily subcutaneous injections of d-galactose over a 6-week period [54]. In that study, aminoguanidine decreased the abnormally elevated levels of total testosterone and serum AMH [54,98]. In another experiment by He *et al.* [99] the inhibitory effect of aminoguanidine on dietary AGEs bioactivity in Sprague Dawley rats was studied and they demonstrated a marked increase in urinary secretion of AGEs as well as reduction in the deposition of dietary AGEs in kidneys and liver. AST-120, is an oral adsorbent that binds with CML, a food derived AGEs, thus reducing its gastrointestinal absorption [94]. Although AST-120 has never been studied in PCOS setting, the intake of AST-120 for 3 months reduced serum AGEs levels and downregulated RAGE mRNA levels in non-diabetic patients with chronic renal failure [97].

The therapeutic effect of metformin, an insulin-sensitizer drug, in improving the metabolic and hormonal profiles in PCOS has been well established [95]. One of the mechanism by which metformin acts is by converting MG to a less potent substance such as dihydroimidazolone ultimately reducing oxidative stress [95,100]. In women with PCOS, metformin leads to a drop in testosterone levels and free androgen index without significant change in metabolic parameters such as BMI [95]. Metformin can also reduce expression and prevent up-regulation of RAGE, which can prevent AGE-induced cell damage [101,102]. In women with PCOS, Diamanti-Kandarakis *et al.* [95] demonstrated that metformin intake for 6 months led to a significant reduction in serum AGE levels. The decrease in serum AGEs levels was more prominent in women with PCOS who had normal glucose tolerance (*p* = 0.02) as compared to those who had abnormal glucose intolerance [95].

Orlistat, a lipase inhibitor, also reduces post-meal AGEs’ levels in patients with PCOS along with inhibition of fasting insulin and testosterone concentrations [96]. Diamanti-Kandarakis E *et al.* [103] investigated the effect of post meal orlistat in 14 healthy and 10 women diagnosed with type 2 diabetes who were given high-AGE diet for two days. The data resulted in acutely decreased absorption of AGEs after orlistat intake in control group with no apparent benefit in diabetic group [103]. In another study, the investigators found significant reduction in serum AGEs’ level in women with PCOS after treatment with orlistat for 6 months [96]. The same authors reported significantly lower serum AGEs levels after administration of high-AGE diet combined with orlistat in comparison to high-AGE diet only in women with PCOS [104].

**Table 2:** Methods for attenuating the effect of AGEs.

Methods	Effect on AGEs
1. Change in the food preparation methods	
Food preparation at low temperature with high moisture with brief heating time	Reduce the dietary intake of AGEs
Use of acidic marinades such as lemon juice and vinegar	
2. Vitamin D supplementation	Elevate serum sRAGE levels in women with PCOS
3. Oral adsorption of dietary AGEs	
Aminoguanidine	Increase urinary secretion of AGEs
	2. Decrease deposition of AGEs in kidney and liver
AST-120	Reduce serum AGEs’ levels Reduce RAGE mRNA expression levels
4. Insulin sensitizer	
Metformin	Downregulation of RAGE in osteoblast-like cells
	2. Decrease serum AGEs’ levels in women with PCOS
5. Lipase inhibitor	Reduce post-meal serum AGEs’ levels
Orlistat	
6. Alpha-lipoic acid (ALA)	Reduce formation of AGEs
7. Pyridoxamine	Reduce formation of AGEs

Some other compounds such as angiotensin-converting enzyme inhibitors or angiotensin II type 1 receptor blockers, pentoxyfylline, desferoxamine, penicillamine, aspirin, vitamin C or E (antioxidants), amadoriases (enzymes that causes degradation of amadori products), phenacylthiazolium bromide (disrupt α-dicarbonyl cross-link), aryl ureido, and aryl carboxaminido phenoxy isobutyric acids derivatives are being investigated as AGE inhibitors [105,106,107,108,109].

## 8. Conclusions

Dietary AGEs are pathologic substances that have been implicated in the development and progression of various metabolic and chronic diseases, and more recently PCOS. Thus, it is reasonable to speculate that significantly reduced intake of diet containing AGEs has favorable effects on the metabolic and hormonal profiles as well as ovarian function in women PCOS. Further studies are needed to establish an optimal low-AGE diet in order to prevent and/or treat the hormonal imbalance and the ovulatory dysfunction observed in women with PCOS. Modifying food preparation methods with the aim of containing the least amount of AGEs could potentially improve ovulatory dysfunction associated with PCOS.
